# Diagnosis and Stratification of *Pseudomonas aeruginosa* Infected Patients by Immunochemical Quantitative Determination of Pyocyanin From Clinical Bacterial Isolates

**DOI:** 10.3389/fcimb.2021.786929

**Published:** 2021-12-14

**Authors:** Barbara Rodriguez-Urretavizcaya, Nuria Pascual, Carme Pastells, Maria Teresa Martin-Gomez, Lluïsa Vilaplana, Maria-Pilar Marco

**Affiliations:** ^1^ Nanobiotechnology for Diagnostics (Nb4D), Institute of Advanced Chemistry of Catalonia, Institute for Advanced Chemistry of Catalonia (IQAC)-Spanish National Research Council (CSIC), Barcelona, Spain; ^2^ Centro de Investigación Biomédica en Red (CIBER) de Bioingeniería, Biomateriales y Nanomedicina (CIBER-BBN), Madrid, Spain; ^3^ Microbiology Department, Hospital Universitari Vall d’Hebron, Barcelona, Spain

**Keywords:** quorum sensing, pyocyanin, ELISA, *Pseudomonas aeruginosa*, monoclonal antibody, diagnostic

## Abstract

The development of a highly sensitive, specific, and reliable immunochemical assay to detect pyocyanin (PYO), one of the most important virulence factors (VFs) of *Pseudomonas aeruginosa*, is here reported. The assay uses a high-affinity monoclonal antibody (mAb; C.9.1.9.1.1.2.2.) raised against 1-hydroxyphenazine (1-OHphz) hapten derivatives (PC1; a 1:1 mixture of 9-hydroxy- and 6-hydroxy-phenazine-2-carobxylic acids). Selective screening using PYO and 1-OHphz on several cloning cycles allowed the selection of a clone able to detect PYO at low concentration levels. The microplate-based ELISA developed is able to achieve a limit of detection (LoD) of 0.07 nM, which is much lower than the concentrations reported to be found in clinical samples (130 μM in sputa and 2.8 μM in ear secretions). The ELISA has allowed the investigation of the release kinetics of PYO and 1-OHphz (the main metabolite of PYO) of clinical isolates obtained from *P. aeruginosa*-infected patients and cultured in Mueller–Hinton medium. Significant differences have been found between clinical isolates obtained from patients with an acute or a chronic infection (~6,000 nM vs. ~8 nM of PYO content, respectively) corroborated by the analysis of PYO/1-OHphz levels released by 37 clinical isolates obtained from infected patients at different stages. In all cases, the levels of 1-OHphz were much lower than those of PYO (at the highest levels 6,000 nM vs. 300 nM for PYO vs. 1-OHphz, respectively). The results found point to a real potential of PYO as a biomarker of *P. aeruginosa* infection and the possibility to use such VF also as a biomarker for patient stratification[2] and for an effective management of these kinds of infections.

## 1 Introduction

*Pseudomonas aeruginosa* is a common Gram-negative opportunistic multidrug-resistant pathogen that causes acute and chronic infections especially in immunocompromised patients (Barbier et al., 2013; Pieters et al., 2019). It is one of the main pathogens causing nosocomial infections such as hospital-acquired pneumonia (HAP), health care-associated pneumonia (HCAP), ventilator-associated pneumonia (VAP), and ventilator-associated tracheobronchitis (Williams et al., 2010), contributing to a high mortality and morbidity (Gaynes et al., 2005; Sadikot et al., 2005; Pang et al., 2019). It colonizes different parts of the body such as the skin, heart, urinary tract, ears, eyes, airways, and lung tissues, causing urinary infections, burn, respiratory infections, and septicemia (Yang et al., 2011; Ruffin and Brochiero, 2019). Moreover, it is one of the most predominant bacteria in the lungs of patients with cystic fibrosis (CF) (Maurice et al., 2019). CF is an autosomal recessive genetic disease frequent in the Caucasian population caused by mutations in the cystic fibrosis transmembrane conductance regulator (*CFTR*) gene. This gene codes for the CFTR protein that is responsible for maintaining epithelial surface hydration by regulating ion and water transport (De Boeck, 2020). Defective expression of CFTR induces mucus hypersecretion that obstructs airways and ultimately triggers morbidity and mortality in CF patients (Puchelle et al., 2002).

In the last years, as many other bacterial species (Blair et al., 2015), *P. aeruginosa* has developed resistance to antibiotics mainly due to its high adaptability and metabolic versatility (Breidenstein et al., 2011; Klockgether et al., 2011). Usually, *P. aeruginosa* infections are categorized as acute and chronic. The first is associated with a planktonic lifestyle, and it is frequent during early stages of infection. It shows a high virulence factor (VF) expression [as it is the case for pyocyanin (PYO) (Chandler et al., 2019)] and, in general, is susceptible to antibiotics. In contrast, chronic infections are characterized by low VF levels and are more resistant to antibiotics mainly due to biofilm formation (Heacock-Kang et al., 2017) and persistent cell generation (chronicity) (Van den Bergh et al., 2017), both characteristics of resistance mechanisms in *P. aeruginosa* (Taylor et al., 2014; Valentini et al., 2018; Lozano et al., 2018). Therefore, eradication therapies at early stages of the infection are recommended, since at this stage, bacterial strains are more susceptible to antibiotics (Zegans et al., 2002; Boucher et al., 2009; Hirsch and Tam, 2010; Park et al., 2012; Pang et al., 2019).

Plate cultures inoculated from swab samples continue to be the most common practice for *P. aeruginosa* identification, but it can take between 24 and 48 h (Sismaet et al., 2017), which usually results in a delay on the administration of the correct treatment, aggravating the symptoms and/or increasing resistance problems. Hence, the development of efficient tools for early diagnosis could significantly improve the management of *P. aeruginosa* infections. In this context, the identification of new biomarkers of infection has become an essential milestone. The development of pathogenesis and the transition between acute and chronic infection stages are regulated by a bacterial gene regulation mechanism called quorum sensing (QS) system. QS coordinates the expression of a myriad of genes in response to the presence of small signal molecules known as autoinducers (AIs) (Valentini et al., 2018). When a threshold concentration of AIs is reached, the expression of genes that regulate the secretion of VFs and biofilm formation is triggered. Therefore, QS has attracted attention as a promising target to develop diagnostic and therapeutic approaches (Fong et al., 2018; Rehman and Leiknes, 2018).

**Figure 1:**
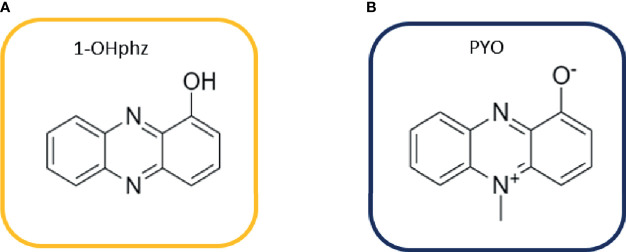
Structures of **(A)** 1-hydroxyphenazine (1-OHphz) and **(B)** pyocyanin (PYO) phenazines. Both molecules are structurally similar having the PYO molecule just an extra methyl group at position 5.

Here, we focus on the main QS-regulated VF of *P. aeruginosa* called PYO, which is specific for this bacterium. PYO is a nitrogen-containing aromatic blue pigment belonging to the family of phenazines that presents unique redox properties (Dietrich et al., 2006; Jayaseelan et al., 2014; Hall et al., 2016). Phenazines exert a large number of effects on host cells such as cytokine production alteration [interleukin (IL)-8 and IL-6 increase], reactive oxygen species production (oxidative stress), cell signaling disruption, and ciliary motion inhibition (Ran et al., 2003; Hall et al., 2016). Moreover, phenazines can cause toxic effects or benefit other cells using electron transfer mechanisms (Costa et al., 2015). They are secreted at high concentrations during early colonization to establish infection (acute infections); however, during chronic infections, their levels are downregulated (D'Argenio et al., 2007; Mena et al., 2008). Apart from absorbance, electrochemical methods (Chen et al., 2015; Sismaet et al., 2017; Sakamoto et al., 2018) and surface-enhanced Raman spectroscopy studies (SERS) (Zukovskaja et al., 2017) are the most commonly used techniques for PYO detection in biological samples. Routine implementation of these technologies for biomedical and clinical analysis is difficult; in contrast, immunochemical analytical methods are positioned as reliable, efficient, and low-cost clinical diagnostic alternatives, reaching low limits of detection (LoDs) and excellent specificity (Krogsrud and Koivunen, 2006). These methods, based on the specific interaction between an antigen and an antibody (Ab), are widely implemented in clinical laboratories (Ma et al., 2019; Vatankhah et al., 2019; Zelini et al., 2019). Few years ago, we reported for the first time the development of polyclonal antibodies (pAbs) and microplate-based ELISA for 1-hydroxyphenazine (1-OHphz), the main metabolite of PYO, with an excellent detectability (IC_50_ = 0.53 nM) (Pastells et al., 2016). It has been reported that 1-OHphz also contributes to the virulence of *P. aeruginosa*. Besides the great structural similarities between PYO and 1-OHphz (**Figure 1**), the PYO cross-reacted only by a 0.1% (IC_50_ >800 nM). Hence, PYO quantification required its complete transformation to 1-OHphz prior to the analysis, which was achieved by treating the samples with a strong base. Here, we report the development of monoclonal antibodies (mAbs) showing a much greater affinity for PYO, which have allowed direct quantification of this phenazine in culture broth of clinical isolates without prior transformation into 1-OHphz. The ELISA developed has been used to assess the potential of PYO and 1-OHphz as biomarkers of infection and patient stratification.

## 2 Materials and Methods

### 2.1 General Methods and Instruments

The pH and conductivity of all buffers and solutions were measured with a pH-meter pH 540 GLP and a conductimeter LF 340, respectively (WTW, Weilheim, Germany).

Polystyrene microtiter plates used for the ELISAs were purchased from Nunc (Maxisorp, Roskilde, Denmark). Dilution plates were purchased from Nirco (Barberà del Vallés, Spain). Washing steps performed during the ELISA were carried out on a Biotek ELx465 (Biotek Inc.). Absorbances were read on a Thermo Scientific MultiSkan GO (Thermo Fisher Scientific, Waltham, MA, USA) at 450-nm wavelength. The competitive curves shown were obtained using a four-parameter logistic equation using GraphPad Prism 7.0 (GraphPad Software Inc., San Diego, CA, USA). The presented results correspond to the average and standard deviation of at least two well replicates.

### 2.2 Buffers and Bacterial Growth Media

Phosphate-buffered saline (PBS) is 10 mM phosphate buffer with 0.8% saline solution, adjusting the pH to 7.5. PBST (assay buffer) is PBS with 0.05% Tween 20. Also, 10× PBS is PBS 10 times concentrated. Coating buffer is 50 mM carbonate-bicarbonate buffer pH 9.6. Citrate buffer is a 40-mM solution of sodium citrate pH 5.5. The substrate solution contains 0.01% TMB (3,3′,5,5′-tetramethylbenzidine) and 0.004% H_2_O_2_ in citrate buffer. Mueller–Hinton (MH) broth (Ref. 70192, Sigma Aldrich) was prepared by diluting 21 g of the corresponding powder in 1 L of deionized water, as indicated by the manufacturer. The resulting liquid medium was subsequently autoclaved.

### 2.3 Chemicals and Immunochemicals

PYO was obtained from Sigma-Aldrich Co. (St. Louis, MO), and 1-OHphz was synthesized following the procedures (Vivian, 1956). The development of the anti-1-OHphz pAb As230 was described by Pastells et al. (2016). The preparation of the PYO mAb C.9.1.9.1.1.2.2. has been performed with the support of the U2 [Custom Antibody Service (CAbS) of the ICTS “NANOBIOSIS”]. PC1-HCH and PC1-BSA bioconjugates were obtained following the same synthetic route described in Pastells et al. (2016).

### 2.4 Preparation of Monoclonal Antibodies

BALB/c female mice (8–10 weeks old) were immunized with PC1-HCH maleic acid (MA). The first dose consisted of 100 µg of bioconjugate injected intraperitoneally as an emulsion of PBS and complete Freund’s adjuvant. In addition, three booster injections were given at 3-week intervals using the same dose of immunogen emulsified in incomplete Freund’s adjuvant. Mice selected as spleen donors for hybridoma production received a final injection of 100 µg of antigen in PBS 4 days prior to fusion.

P3-X63/Ag 8.653 murine myeloma cells (ATCC, Rockville, MD) were cultured in Dulbecco's Modified Eagle Medium (DMEM) (high-glucose DMEM with 2 mM alanylglutamine, 1 mM MEM nonessential amino acids, and 25 μg/ml gentamicin) supplemented with 10% (v/v) fetal bovine serum (FBS). Mouse spleen lymphocytes were fused with myeloma cells at a 4:1 ratio using PEG 1500 (Roche Applied Science, Mannheim, Germany) as a fusing agent. The fused cells were cultured in 96-well culture plates at a density of 2 × 10^5^ cells/100 μl of 15% FBS-supplemented DMEM per well. After 24 h, Hypoxanthine-aminopterin-thymidine medium (HAT) selection medium [10% FBS-supplemented DMEM with 100 μM hypoxathine, 0.4 μM aminopterine, 16 μM thymidine, 2% Hybridoma Fusion and Cloning Supplement (HFCS; Roche)] was added (100 μl/well). Subsequently, 10 days after cell fusion, culture supernatants were screened by performing indirect ELISA assays, coating the corresponding plates with 1.0 μg/ml PC1-BSA conjugate in order to select the hybridomas that were able to recognize PYO with high affinity. The chosen hybridomas were then cloned by the limiting dilution method using HT medium (HAT medium without aminopterine), supplemented with 15% FBS and 1% HFCS. Finally, stable antibody-producing clones were expanded and cryopreserved in liquid nitrogen, and the resulting supernatants containing mAbs were purified by protein G affinity chromatography.

Protocols and procedures have been approved by the CID-CSIC Ethical Committee (local institution), CSIC-CEEA: Ethics Committee for Animal Experimentation of CSIC (CSIC animal experimentation ethical committee, evaluating procedures at the national level), and Catalonian authorities.

### 2.5 Two-Dimensional Experiments

The appropriate dilutions of mAb and PC1-BSA coating antigen required to perform indirect competitive ELISAs were established by carrying out a 2D checkerboard titration assay (Barcelo et al., 1997). This 2D experiment consists of testing serial dilutions of PC1-BSA bioconjugate and mAb in order to select their optimal combination to achieve high absorbance values without reaching the saturation levels of the curve.

### 2.6 Competitive Microplate-Based ELISA

#### 2.6.1 Pyocyanin ELISA

Microtiter plates were coated with PC1-BSA (0.125 μg ml^−1^ in coating buffer 100 μl well^−1^) and incubated overnight at 4°C. Then, the plates were washed four times with PBST (4 × 300 μl) and serial dilutions of PYO (from 3,200 to 0 nM in PBST) or the samples, diluted with the assay buffer, were added (50 μl well^−1^) followed by the solution of the mAb C.9.1.9.1.1.2.2. (0.01575 μg ml^−1^ also in PBST, 50 μl well^−1^). After 30-min incubation shaking at room temperature (RT), the plates were washed as described above, and the anti-immunoglobulin G (anti-IgG)-horseradish peroxidase (HRP) solution (1/2,000 in PBST, 100 μl well^−1^) was added, waiting an additional 30-min incubation period. Subsequently, another cycle of washes was performed, and the substrate solution was added (100 μl well^−1^). Finally, the enzymatic reaction was stopped after 30 min at RT with 2 M H_2_SO_4_ (50 μl well^−1^). The absorbances were measured at 450 nm.

#### 2.6.2 1-Hydroxyphenazine ELISA

Briefly, the procedure used was similar as the PYO ELISA described above but coating the microplates with PC1-BSA at 0.0625 μg ml^-1^ and using the pAb As230 (1/6,000 in PBST) and anti-IgG-HRP (1/6,000 in PBST) (Pastells et al., 2016).

### 2.7 Matrix Effect Studies

Nonspecific interferences produced by bacterial isolates medium were analyzed by preparing standard curves of PYO and 1-OHphz directly in MH broth diluted several times (0 and from 1/5 to 1/20) with PBST and measuring them with the PYO and 1-OHphz ELISAs to assess the parallelism with respect to the calibration curves prepared in the assay buffer.

### 2.8 Accuracy Studies

PYO blind spiked samples (ranging from 30 to 0 nM) were prepared in PBST and MH broth and measured with the PYO ELISA to assess assay accuracy.

### 2.9 Clinical Isolate Samples

Thirty-seven bacterial isolates collected at the Microbiology Department of the Vall d’Hebron University Hospital (VHUH, Barcelona, Spain) from patients diagnosed with *P. aeruginosa* acute or chronic infections (**Table 1**) were tested. Well-isolated colonies from primary cultures were selected and stored frozen in glycerol at -20°C until use. PAO1 strain (ATCC 15692) was used as a reference strain.

**Table 1:** Description of the 37 bacterial isolates from patients infected with *P. aeruginosa*.

Number of patient	Isolate Nb4D	Underlying clinical situation
1	PAAI1	Acute nosocomial infection
2	PAAI2	Acute nosocomial infection
3	PAAI3	Intermittent colonization
4	PAAI4	Acute nosocomial infection
5	PAAI5	Acute nosocomial infection
6	PAAI6	Acute nosocomial infection
7	PAAI7	Acute nosocomial infection
8	PAAI8	Acute infection. Lung transplant recipient
9	PAAI9	Acute infection. Lung transplant recipient
20	PACI1	Chronic infection. Non-CF bronchiectasis
21	PACI2	Chronic infection. Non-CF bronchiectasis
22	PACI3	Chronic infection. Non-CF bronchiectasis
23	PACI4	Chronic infection. Non-CF bronchiectasis
24	PACI5	Chronic infection. CF bronchiectasis. Sinusitis episode
25	PACI6	Chronic infection. CF bronchiectasis
26	PACI7	Chronic infection. CF bronchiectasis
27	PACI8	Chronic infection. CF bronchiectasis
–	PAO1^a^	Control strain
10	PAAI10	Acute nosocomial infection
11	PAAI11	Acute nosocomial infection
12	PAAI12	Acute infection. COPD
13	PAAI13	Acute nosocomial infection
14	PAAI14	Acute infection. Lung transplant recipient
15	PAAI15	Acute nosocomial infection
16	PAAI16	Acute nosocomial infection
17	PAAI17	Intermittent colonization. Reagudization
18	PAAI18	Acute infection
19	PAAI19	Acute infection
28	PACI9	Chronic infection. Non-CF bronchiectasis
29	PACI10	Chronic infection. Non-CF bronchiectasis
30	PACI11	Chronic infection. Non-CF bronchiectasis
31	PACI12	Chronic infection. Non-CF bronchiectasis. Hemoptysis
32	PACI13	Chronic infection. Non-CF bronchiectasis
33	PACI14	Chronic infection. CF-bronchiectasis
34	PACI15	Chronic infection. CF-bronchiectasis. Acute viral infection
35	PACI16	Chronic infection. CF-bronchiectasis
36	PACI17	Chronic infection. CF-bronchiectasis
37	PACI18	Chronic infection. CF bronchiectasis. Reagudization

a PAO1 was used as a positive control strain.

The experimentation reported here has been approved by the CEIC (Ethical Committee for Clinical Research) of the Hospital Vall d’Hebron and the ethical committee of CSIC.

### 2.10 Bacterial Isolate Growth Medium and Inoculum Preparation

Bacterial clinical isolate frozen stocks from patients with proven infection by *P. aeruginosa* were inoculated onto Columbia agar plates supplemented with 5% sheep blood (Biomerieux Ref. 43041) and incubated overnight at 37°C. Next day, four or five colonies from each plated isolate were transferred using a sterile swab into 10-ml Falcon with 3-ml MH liquid media (initial suspension) and kept shaking (500 rpm) at 37°C. When turbidity reached values [measured as optical density (OD) at 600 nm] between 0.2 and 0.3 (equivalent to a McFarland turbidity containing approximately 1 × 10^8^ CFUs ml^-1^), sample aliquots (10 μl) were taken and diluted in MH broth (10 ml) at different time intervals (t_0_, start time point) to preserve the conditions and to avoid growth disruption due to changes in the temperature or other eventualities.

### 2.11 Bacterial Growth Measurements and Phenazine Production Kinetics

Bacterial suspension aliquots collected at different incubation times were used to plot bacterial growth curves and to build up PYO and 1-OHphz kinetic production curves. Bacterial growth studies were performed by measuring the turbidity at each time point in broth culture aliquots. Turbidity correlates with cell density during the logarithmic growth phase and was measured as OD at 600 nm. In addition, CFUs were counted by plating serial dilutions of each time point aliquot and counting the colonies after 24-h incubation.

The OD of each sample was measured at 600 nm at RT in a Multiskan GO spectrophotometer using High Precision Cell cuvette made of quartz (Hellma Analytics). CFUs were determined by carrying out serial 1:10 dilutions of each time point aliquot (10 µl) on 5% blood agar plates (37°C, 24 h). In each case, the corresponding dilutions plated were established according to turbidity values (dilutions ranged from 1/10 to 1/10^8^) (Sarkar et al., 2017).

Collected bacterial time point aliquots were centrifuged (500 G, 5 min) for PYO mAb/PC1-BSA ELISA measures. The resulting supernatant was diluted 20 times in assay buffer to avoid matrix effects. For 1-OHphz measurements, a 1/5 dilution in assay buffer was carried out (Pastells et al., 2016).

## 3 Results

### 3.1 ELISA Assays for Phenazines: 1-Hydroxyphenazine and Pyocyanin

A few years ago, we reported the development of an immunochemical assay to quantify PYO in complex biological media (Pastells et al., 2016). For this purpose, we used a 1-OHphz hapten to raise antibodies with the expectation that would recognize also PYO due to the chemical difficulties to obtain a stable PYO hapten. Although PYO and 1-OHphz are structurally closely related molecules (**Figure 1**), the antisera obtained showed much higher avidity for 1-OHphz (IC_50_ = 0.62 nM) than that for PYO (IC_50_ >800 nM; % CR <0.1%). For this reason, PYO quantification required its complete transformation to 1-OHphz, which was achieved by treating the samples with a strong base (Pastells et al., 2016). Aiming at simplifying the procedure, we attempted to accomplish a high-affinity mAb against PYO using the hybridoma technology that allows selecting cell clones with tailored features based on a rational selective screening approach. With this aim, mice were immunized with PC1-HCH (1-OHphz hapten linked to HCH), and the hybridoma cells obtained after the fusion step were selectively screened during several cloning cycles to select those clones that best detected PYO on a competitive format using both phenazines, PYO and 1-OHphz. Afterward, bidimensional checkerboard experiments (2D-ELISAs) allowed to determine the most appropriate concentrations for the bioconjugate competitor PC1-BSA and each mAb to be used on the competitive ELISAs. **Table 2** shows the IC_50_ values of the best selected 14 clones for PYO and 1-OHphz using the developed ELISAs according to the abovementioned criteria. In all cases, final clones presented a better detectability for 1-OHphz than for PYO. While some of them (C.9.1.9.7.3.1.1. and C.9.1.7.2.1.2.1.) showed much higher affinity for 1-OHphz than for PYO (IC_50_ values for PYO ranging 15–20 µM), other clones (C.9.1.4.1.1.4.2. and C.9.1.4.1.1.4.4. C.9.1.9.1.1.2.2.) had more similar selectivities for both phenazines, with low IC_50_ values for PYO (<2 nM). From these three best clones, C.9.1.9.1.1.2.2. (from now on PYO mAb122) was selected to quantify PYO levels in clinical samples, as it showed better ELISA parameters such as slope and Abs_max_ (data not shown).

**Table 2:** Features of the monoclonal antibodies (mAb) selected in this study and of the As230 with respect to the recognition of PYO and 1-OHphz.

Clone name^b^	IC_50_ (1-OHphz)^c^	IC_50_ (PYO)^d^	Relation [(IC_50_ (PYO)/IC_50_ (1-OHphz)]
**C.9.1.7.2.1.2.1**	2.17	16.26	7.48
**C.9.1.7.2.1.2.2**	0.43	3.87	8.99
**C.9.1.9.7.3.1.1**	3.72	27.21	7.31
**C.9.1.9.7.3.1.2**	1.23	7.88	6.42
**C.9.2.8.3.1.2.2**	0.90	5.98	6.67
**C.9.1.7.1.1.2.1**	1.19	7.39	6.22
**C.9.1.4.1.1.4.2**	0.24	1.97	8.37
**C.9.1.4.1.1.4.1**	0.38	3.81	10.13
**C.9.1.9.1.1.2.2**	0.32	2.96	9.27
**C.9.1.4.1.1.4.4**	0.26	2.13	8.06
**C.9.1.4.1.1.4.3**	1.10	6.56	5.99
**C.9.2.8.3.1.2.3**	0.83	7.45	8.96
**C.9.1.7.1.1.1.2**	1.34	9.23	6.91
**C.9.1.7.1.1.2.3**	0.79	5.01	6.37
**As 230 (** Pastells et al., 2016)	0.62	> 800	1290.32

^a^A**_max_** values were between 0.8 and 1.2.

^b^Antibody concentration used 0.008 µg/ml for mAb122 and 1/3,000 dilution for As230.

^c,d^Coating antigen concentration used 0.125 and 0.0625µg/ml for PYO mAb122/PC1-BSA and As230/PC1-BSA, respectively. The IC_50_ values were determined using the protocol of the PYO mAb122/PC1-BSA (see experimental section) and As230/PC1-BSA (Pastells et al., 2016) for the case of As230.

Green means that was the selected clone for developing the ELISA.

The analytical parameters of the PYO mAb122/PC1-BSA ELISA are summarized in **Table 3**. The low LoD (0.07 nM in the assay buffer and 0.15 nM in MH diluted 20 times) reached allowed contemplating the possibility to directly quantify PYO without the need to convert it to 1-OHphz. Even if this last metabolite was much better recognized (IC_50_s: 0.32 vs. 2.92 nM for 1-OHphz and PYO, respectively; **Table S2** and **Figure S3**), it was expected to find it at much lower levels. Hence, Wilson et al. (1988) reported 1-OHphz levels to be much lower (9-fold times lower) than PYO concentration in sputum samples. Nevertheless, in this work, the PYO concentrations measured have been expressed as PYO immunoreactivity equivalents (PYO IRequiv) to take this fact into consideration.

**Table 3:** Features of the PYO mAb122/PC1-BSA ELISA in PBST and MH broth diluted 20 times.

	PBST	MH 1/20
**A_min_**	0.07 ± 0.01	0.05 ± 0.01
**A_max_**	1.08 ± 0.09	0.92 ± 0.14
**Slope**	-1.40 ± 0.49	-1.11 ± 0.21
**IC_50_/nM**	0.68 ± 0.10	1.18 ± 0.24
**Dynamic range**	0.18 ± 0.08 and 2.18 ± 0.19	0.32 ± 0.10 and 4.13 ± 1.35
**LoD**	0.07 ± 0.04	0.15 ± 0.07
**R^2^**	0.99 ± 0.01	0.99 ± 0.01

Assay conditions used were PC1-BSA at 0.125 µg/ml and PYO mAb122 at 0.008 µg/ml in both cases. The data shown correspond to the average of the parameters of the calibration curves performed on three different days using at least three well replicates per concentration.

The robustness of the assay was demonstrated by measuring IC_50_, slope, and Abs_max_ values during a year (n = 13). Thus, the obtained results followed a normal distribution (D’Agostino & Pearson normality test, Shapiro–Wilk normality test, and Kolmogórov-Smirnov (KS) normality test) showing values of 1.72 ± 0.79, -1.28 ± 0.27, and 0.97 ± 0.29, respectively (**Figure S1**). Moreover, the low variability of the assay is demonstrated through the low dispersion of the obtained results, which are concentrated around the mean value. In this sense, IC_50_ values have only oscillated between 0.8 and 2.8, the slope between -0.9 and -1.7, and the Abs_max_ between 0.8 and 1.5 (outliers are not considered). In addition, the assay was able to work between 4.5 and 7.5 pH values, showing slightly better performance at 6.5 and 7.5 values (**Figure S2**) with acceptable IC_50_ values. At pH below 4.5 and over 7.5, there was a drastic decrease observed in the absorbance of the assay.

### 3.2 Implementation of Phenazine ELISAs to the Analysis of Bacterial Isolates

**Figure 2:**
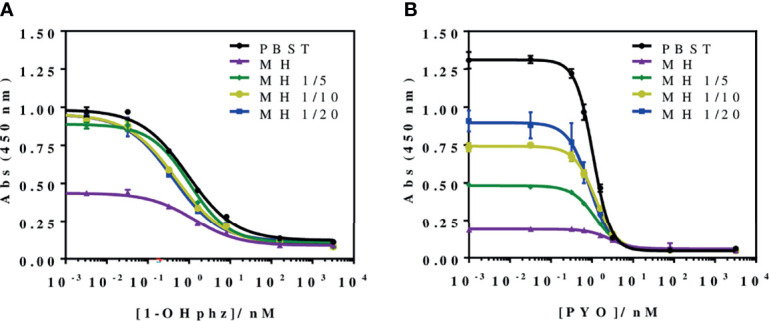
Matrix effect of the Mueller–Hinton (MH) broth undiluted and diluted 5, 10, and 20 times with PBST on the **(A)** PYO mAb122/PC1-BSA and **(B)** As230/PC1-BSA ELISA. The calibration curves were run using the conditions established for the assay in PBST. The results demonstrate that it is possible to perform direct measurements in MH media diluted from 5 to 20 times in PBST for both phenazines (see **Table 3** for analytical parameters of the standard curves in PBST and in 1/20 MH on PYO mAb122/PC1-BSA and **Table 4** for analytical parameters of the standard curves in PBST and in 1/5 MH). The results shown are the average and standard deviations of analysis made by triplicates.

**Figure 3:**
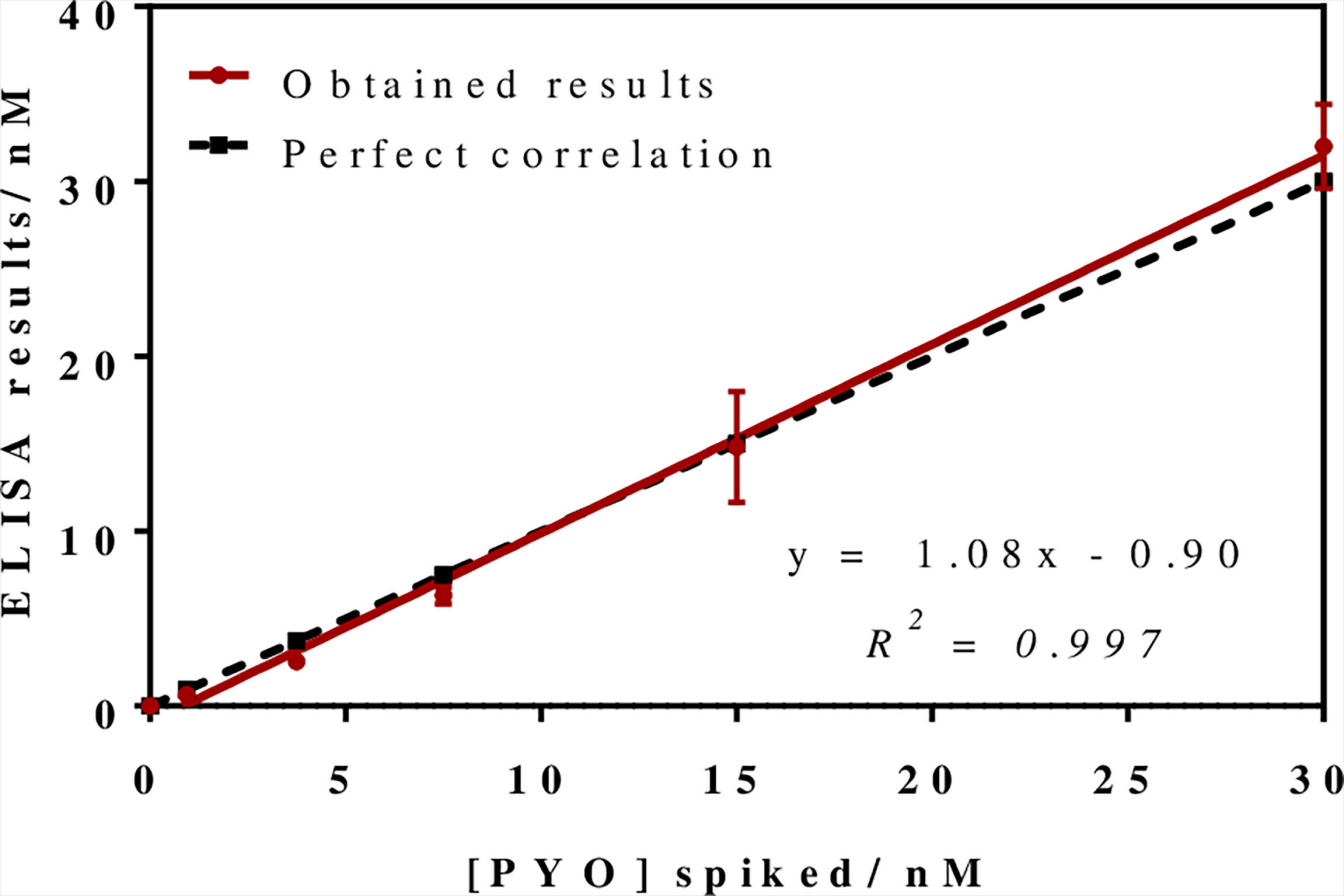
Results from the accuracy study. The graph shows the linear regression analysis obtained representing the different pyocyanin (PYO) concentrations spiked in Mueller–Hinton (MH) broth against the concentration measured with the PYO mAb122/PC1-BSA ELISA. Assays were run in MH culture media diluted 1/20 using PBST. Each calibration point was measured in triplicates on the same ELISA plate and the results show the average and standard deviation of analysis made on three different days.

To investigate the release profile of both PYO and 1-OHphz by different clinical bacterial isolates obtained from patients infected with *P. aeruginosa* at different stage, clinical isolates were grown in MH broth. Sample aliquots were taken at different times to be measured with PYO mAb122/PC1-BSA ELISA reported here and with 1-OHphz As230/PC1-BSA ELISA already published (Pastells et al., 2016). Before, the potential nonspecific interferences caused by the MH broth in the ELISA were initially assessed. For this purpose, calibration curves of PYO and 1-OHphz were prepared in MH diluted at different ratios with PBST (from 1/5 to 1/20) and measured with the corresponding ELISA to compare their performance with the calibration curves run in buffer. As observed in **Figure 2**, PYO mAb122/PC1-BSA ELISA worked very well in MH broth after just a 1/5 dilution (IC_50_ = 1.22 nM), although the signal was significantly decreased under these conditions. However, considering that the detectability achieved by this ELISA was well below the values reported in clinical samples (μM range) (Hall et al., 2016), we decided to attempt measuring the samples using a 1/20 dilution of MH in PBST to investigate the release profile of the abovementioned clinical bacterial isolates. As shown in **Table 3**, the analytical parameters of the ELISA run in 1/20 MH seemed to be suitable for our purposes (IC_50_ = 1.18 ± 0.24, LoD = 0.15 ± 0.07). Moreover, accuracy studies performed under these conditions supported this fact. As observed in **Figure 3**, the coefficient of correlation between concentrations of the blind spiked samples and the values measured with the ELISA was excellent (R^2^ = 0.997), and the slope of the linear regression was close to 1 (slope = 1.08), showing almost a perfect match between spiked and measured concentration values. In the same line, calibration curves of 1-OHphz were prepared in different dilutions of MH in PBST (from 1/5 to 1/20; **Figure 2**) and evaluated using As230/PC1-BSA ELISA. The obtained results showed that the assay could be used to measure MH samples diluted just five times with the assay buffer (IC_50_ = 1.02 nM). The analytical parameters of the ELISA run in 1/5 MH are illustrated in **Table 4**.

**Table 4:** Features of the As230/PC1-BSA ELISA in PBST and MH broth diluted five times.

	PBST	MH 1/5
**A_min_**	0.12 ± 0.02	0.11 ± 0.02
**A_max_**	0.99 ± 0.02	0.89 ± 0.02
**Slope**	-0.75 ± 0.07	-0.90 ± 0.11
**IC_50_/nM**	0.93 ± 0.05	1.02 ± 0.06
**Dynamic range**	0.17 and 6.47	0.24 and 5.85
**LoD**	0.06	0.10
**R^2^**	0.99 ± 0.03	0.99 ± 0.02

Assay conditions used were PC1-BSA at 0.0625 µg/ml and 1/6,000 dilution of As230 in both cases. The data shown correspond to the average of the parameters of the calibration curves performed on the same day using at least three well replicates per concentration.

### 3.3 Growth Curves and Phenazine Release Profile of *P. aeruginosa* Bacterial Isolates

Bacterial growth curves were built by culturing some clinical isolates from patients with acute and chronic infections (**Table 1**). These measurements were important to compare growth dynamics of the different clinical isolates and also to determine the time required to detect these phenazines in the media using the ELISA developed. Thus, in total, five *P. aeruginosa* isolates (using PAO1 as reference) were incubated in MH media to study their growth dynamics through the measurement of culture turbidity (OD 600 nm) and CFU counting (**Table S1**).

Recently, we have reported that the levels released by clinical isolates of *P. aeruginosa* AIs of the *pqs* QS system, such as PQS and HHQ, were significantly different depending on the stage of the infection (Montagut et al., 2020; Montagut et al., 2021). Thus, for the case of bacterial isolates from patients with acute infection, quantifiable PQS levels could be measured after 5-h growth, while those belonging to patients with a chronic infection, levels could only be quantified after more than 12-h growth, which was correlated to the virulence and behavior of *P. aeruginosa* due to their high adaptability to the environment.With this scenario, we were expecting to find similar differences in terms of the release of a QS-regulated VF such as PYO.

**Figure 4:**
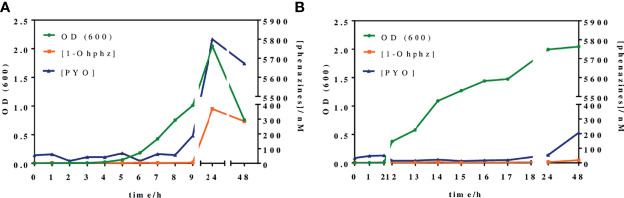
Growth curves and phenazines kinetics production of two *P. aeruginosa* isolates grown for 48 h. The green line (OD 600) gives information about the bacterial growth. The blue and the orange lines indicate IRequiv. of pyocyanin (PYO) and 1-hydroxyphenazine (1-OHphz) production, respectively. Results obtained **(A)** from an isolate (PAAI20) of a patient undergoing an acute *P. aeruginosa* infection and **(B)** from an isolate (PACI18) of a patient suffering chronic infection. The data shown are the average and standard deviation of IRequiv. of PYO and 1-OHphz determinations performed with the corresponding ELISAs on the same day using three well replicates.

**Figure 4** shows the growth curves obtained when analyzing two representative bacterial isolates, PAACI18 and PAAI20, from patients with a chronic and an acute infection, respectively. As expected, a clear difference between them was observed. Thus, the PAAI20 isolate started its exponential growth phase after 6 h of incubation, whereas PAACI18 showed a longer lag phase, initiating its exponential division at 12 h of incubation, in agreement with the behavior observed for the QS signaling molecules of these same types of bacterial isolates. Moreover, the growth profile of both isolates reached the stationary phase. However, in the case of PAAI20, after getting to the maximum growth levels, the curve showed a very steep drop caused by the cytotoxic effect of PYO high levels (it should be noticed that after 24 h of incubation, bacterial cultures corresponding to isolates from acute infections show a clear blue color) (D'Argenio et al., 2002; Hall et al., 2016). Focusing on CFU counting, both isolates reached a plateau on the number of viable cells, similar regarding the values obtained but the isolate PAAI20 yielded the maximum cell number at 7 h of incubation and, by contrast, the isolate PACI18 hit this point after 15 h of culturing time (**Table S1**).

In parallel, at the same time points, bacterial culture aliquots were also analyzed to determine the pattern of phenazine production kinetics of *P. aeruginosa* isolates obtained from acute and chronic infections. The results have been plotted in the same graphs of **Figure 4**. As observed, the release kinetics of the two phenazines studied are coherent with the results previously reported by Montagut et al. (2020, 2021) for the QS signaling molecules mentioned above. Thus, the bacterial isolate from the patient with an acute infection clearly shows much higher levels of both molecules (5,800 nM for PYO and 370 nM for 1-OHphz at 24 h) than the isolate obtained from a patient with chronic infection (54 nM for PYO and 5 nM for 1-OHphz at 24 h).

Focusing on the phenazine production kinetic patterns found, they remain constant when studying an isolate from a patient suffering from a chronic infection, just slightly increasing for PYO after 48 h of culturing. In contrast, the phenazine production profile of an isolate from a patient with an acute infection shows a marked increase in PYO levels, which reaches its maximum at 24 h of incubation, decreasing smoothly after 48 h of growth predictably due to the effects of PYO itself on bacterial viability causing autolysis (Meirelles and Newman, 2018; Ahmed et al., 2019). Furthermore, in all cases, both phenazines showed the same secretion profile, being the levels of the precursor 1-OHphz much lower than those determined for the VF PYO. As an example, at the highest point of the growth curve of the clinical isolate from the patient with an acute infection, PYO reached values near 6,000 nM, while the highest level of 1-OHphz achieved was around 400 nM.

In light of these results, we decided to expand our studies, analyzing the release of PYO and 1-OHphz from 37 bacterial isolates obtained from patients with different respiratory infections at different stages and with distinct symptom severity. The clinical isolates were grown for 16 h to ensure measurements, in case the isolates would belong to a patient with a chronic infection. As a reference control, the strain PAO1 was also cultured under the same conditions, and the phenazines produced by it were also quantified.

**Figure 5:**
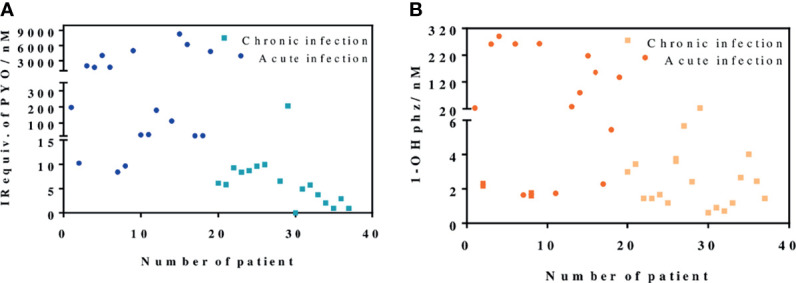
PYO IRequiv. **(A)** and 1-hydroxyphenazine (1-OHphz) levels **(B)** (expressed in nM) found in the Mueller–Hinton (MH) media where clinical bacterial isolates obtained from patients infected with *P. aeruginosa* were growth during 16 h. Dark blue and orange dots correspond to levels from isolates of patients with acute infection and light blue and yellow dots from patients with chronic infection. Each data point represents the average and standard deviation of the results obtained on experiments performed on the same day using three well replicates. See **Table 1** for information on the clinical record of the patients.

As shown in **Figure 5**, the data obtained confirmed the differences found in the behavior of clinical isolates from acute and chronic infections regarding the levels of PYO and 1-OHphz released. Moreover, with few exceptions, which could be due to clinical casuistry, the levels of both phenazines were remarkably higher. Most values found in the MH samples from clinical isolates from patients diagnosed with an acute infection caused by *P. aeruginosa* were between 6,000 and 200 nM for PYO and between 200 and 20 nM for 1-OHphz (**Table S2**). Conversely, they were much lower when the phenazine levels were determined in the culture broth samples of clinical isolates from patients suffering chronic infection. In this case, they were in the low nM range; most values found were between 15 and 1 nM for PYO and between 4 and 0.5 nM for 1-OHphz (**Table S2**). For all clinical isolates, it could be confirmed that the PYO levels are higher than those of the precursor 1-OHphz.

## 4 Discussion

*P. aeruginosa* is considered one of the most threatening pathogens worldwide given its multidrug-resistant and adaptability behavior. It causes a wide variety of infections predominantly in individuals with other diseases. *P. aeruginosa* infections are commonly classified as acute and chronic infections according to the infection status and characteristics (Furukawa et al., 2006). Due to its highly adaptability, this bacterium can survive for long periods of time in the lungs of patients (chronicity) in the form of biofilm (mucoid phenotype). This causes resistance processes, making antibiotic therapies less effective. Therefore, it is essential to have appropriate diagnostic strategies to detect infections at early stages (acute infections). This early detection will allow applying more specific antibiotic treatments, preventing chronification of the infections and the appearance of resistant strains (Vilaplana and Marco, 2020).

It has been already reported that when *P. aeruginosa* changes to a mucoid phenotype (chronic infection), exopolysaccharide production is increased, whereas motility and VF production are decreased (Smith et al., 2006; D'Argenio et al., 2007; Ryall et al., 2014). The immunochemical assay described here has allowed the measurement of levels of the PYO VF in bacterial isolates obtained from patients undergoing different stages of *P. aeruginosa* infections with high sensitivity (LoD at low nM range). All the data obtained have reinforced the hypothesis of PYO as a potential biomarker for discerning among acute and chronic *P. aeruginosa* infections.

The most commonly used methods for PYO detection are based on its unique optical and electrical properties. In this sense, PYO can be detected by absorbance at different wavelengths (Reszka et al., 2004), UV-Vis or mass spectroscopy (MS) (Wilson et al., 1988), SERS (Polisetti et al., 2017), or electrochemical systems (Alatraktchi et al., 2018) reaching LoDs in the low µM and in the high nM range. However, some of these techniques lack specificity (i.e., UV-Vis), may take from 24 to 48 h to render results, or require expensive equipment and need time-consuming pretreatment steps. In contrast, the immunochemical assay described here achieves a very low LoD (low nM range) and does not require any sophisticated equipment. Previously, we reported a microplate-based ELISA (Pastells et al., 2016) able to detect PYO with an LoD of 0.01 nM in less than 90 min. However, this ELISA uses polyclonal antisera showing a much greater avidity for 1-OHphz than to PYO, which required prior transformation of PYO to 1-OHphz. In contrast, the immunochemical strategy reported in this paper is the result of using the hybridoma technology to select a mAb cell clone able to recognize PYO with a much higher affinity. This mAb has been obtained by immunizing mice with the same hapten described by Pastells et al. (2016) derived from the more stable 1-OHphz molecule. Performance of selective cloning rounds allowed to isolate 14 clones that were highly sensitive to PYO. Although all those clones detected 1-OHphz with higher avidity than PYO, three clones showed similar IC_50_ for both phenazines. Hence, clone C.9.1.9.1.1.2.2. (mAb122) was selected to determine PYO levels on clinical isolates. Due to the specific interference caused by 1-OHphz, PYO concentrations are expressed as IRequiv of PYO. Moreover, with the aim of analyzing 1-OHphz quantity on the culture media and determine its contribution, As230/PC1-BSA ELISA was used (Pastells et al., 2016).

The low LoD (0.04 nM) achieved using mAb122 allowed the direct measurement of PYO in culture broth where bacterial isolates from patients infected with *P. aeruginosa* were grown after inoculation of bacteria. Furthermore, the obtained LoD and dynamic ranges may allow the direct quantification of PYO in different clinical samples such as ear secretions (up to 8.1 µM using HPLC-UV) (Reimer et al., 2000), wounds (up to 2.8 µM using chloroform and acid extraction) (Cruickshank and Lowbury, 1953), and sputa (up to 100 µM using HPLC-UV) (Wilson et al., 1988) without any previous treatment or chemical transformation step.

In this paper, we have used the PYO mAb122/PC1-BSA here reported and the As230/PC1-BSA (Pastells et al., 2016) ELISAs to quantify PYO and 1-OHphz levels released by bacterial isolates obtained from patients infected with *P. aeruginosa*. Both ELISAs, showing LoDs in the low nM range, have proven to be robust under different physicochemical conditions including the analysis of a complex biological sample such as the MH broth used to grow the clinical isolates. Thus, bacterial isolates from patients with acute infections released higher concentrations of PYO and 1-OHphz (from 100 to 6,000 nM and from 20 to 100 nM, respectively) than those with chronic infections (5–8 nM and 1–3 nM, respectively). In light of these results, it seems clear that the concentration of phenazines released is correlated with the type or stage of *P. aeruginosa* infection. Moreover, these results are consistent with previously reported studies (Heacock-Kang et al., 2017; Faure et al., 2018; Lozano et al., 2018; Chandler et al., 2019) that showed how, during early stages of *P. aeruginosa* infection, VF and exoproduct expression is increased (acute infections), whereas at chronic stages, *P. aeruginosa* adapts to the host environment by reducing bacterial invasiveness, which means decreasing the expression of toxins, VFs, and QS molecules. In that respect, the described PYO mAb122/PC1-BSA ELISA could be a valuable technique to understand the stage of the disease and to provide patients with the most appropriate treatment strategy.

Bacterial cultures are confined systems where, in addition to the culture components, VFs, proteins, and other bacterial exoproducts are released. Therefore, these culture media are quite complex samples, which predict further successful implementation of the technology here reported to the direct analysis of clinical samples such as sputum, bronchoalveolar lavage (BAL), urine, or plasma. The preliminary data presented point out the possibility to use the reported PYO ELISA to diagnose *P. aeruginosa* infections in clinical samples and to help to stratify patients according to the nature of their infection stage (chronic or acute) based on PYO content. This positions PYO as a potential useful biomarker of *P. aeruginosa* infections. Further investigations may open new avenues of knowledge resulting from the possibility to monitor PYO levels in clinical samples, such as the possibility to predict exacerbations or to diagnose the infection at early stages of the diseases. Moreover, the early and rapid detection of *P. aeruginosa* could reduce the morbidity and mortality of infected patients by giving them a more specific treatment. This will avoid chronification, minimizing resistance to antibiotics and, in consequence, improving the patient’s quality of life. In fact, the described immunochemical approach could be used on different analytical configurations, such as point-of-care (PoC) immunosensor devices, to rapidly detect *P. aeruginosa*.

In the same line, the ineffectiveness of classic antibiotics as a consequence of their inappropriate use has resulted in the rise of *P. aeruginosa* multidrug-resistant strains. However, patients are still treated with the same classical antibiotics, especially β-lactam antibiotics alone and in combination with other families of these compounds due to the lack of new therapeutic approaches (O'Donnell et al., 2020). Among the new emerging therapeutic techniques, mAbs are gaining importance as a consequence of their high affinity and specificity, which minimize possible side effects (Ducancel and Muller, 2012; Khan et al., 2017). Moreover, mAb market has doubled in size in the last 5 years (Grilo and Mantalaris, 2019). Thus, mAbs are increasingly used in different human diseases such as infectious diseases; currently, 4 mAbs are approved by the US Food and Drug Administration (FDA) for this purpose (Lu et al., 2020). In this sense, the here described mAb against PYO could also be tested as a therapeutic agent, since it could minimize or even avoid the cytotoxic effects produced by PYO VF (Ran et al., 2003; Hall et al., 2016) by specifically binding it.

## Data Availability Statement

The raw data supporting the conclusions of this article will be available by the authors without undue reservation if required.

## Ethics Statement

The experimentation reported here has been approved by the CEIC (Ethical Committee for Clinical Research) of the Hospital Vall d’Hebron and the Ethical Committee of CSIC. Written informed consent to participate in this study was provided by the participants’ legal guardian/next of kin. Animal study protocols and procedures have been approved by the CID-CSIC Ethical Committee (local institution), CSIC-CEEA (CSIC animal experimentation ethical committee, evaluating procedures at national level), and Catalonian authorities.

## Author Contributions

BR-U and LV planned and designed the experiments. BR-U carried out all the experiments, performed the analysis and statistics of the obtained results, and wrote the final article. PC designed the 1-OHphz hapten and developed the As230/PC1-BSA ELISA. NP helped in the synthesis of the here presented antibodies. MT-M provided the clinical isolates from patients infected with *P. aeruginosa*. LV and M-PM supervised all the work. All authors discussed the results and contributed to the final article. All authors contributed to the article and approved the submitted version.

## Funding

This work has been funded by the Spanish Government to M-PM through the Ministry of Science and Innovation (SAF2015-67476-R, RTI2018-096278-B-C21, PI, M-PM) and by Fundació Marató de TV3 (201825-30-31, PI, M-PM). The Nb4D group is a consolidated research group (Grup de Recerca) of the Generalitat de Catalunya and has support from the Departament d’Universitats, Recerca i Societat de la Informació de la Generalitat de Catalunya (expedient: 2017 SGR 1441). CIBER Actions are financed by the Instituto de Salud Carlos III with assistance from the European Regional Development Fund (ERDF). Moreover, BR-U has an FI fellowship from the AGAUR (Agència de Gestió d’Ajuts Universitaris I de Recerca) of the Government of Catalonia (Generalitat de Catalunya) (2019FI_B00289). *El Fons social Europeu Inverteix en el teu futur*.

## Conflict of Interest

The authors declare that the research was conducted in the absence of any commercial or financial relationships that could be construed as a potential conflict of interest.

## Publisher’s Note

All claims expressed in this article are solely those of the authors and do not necessarily represent those of their affiliated organizations, or those of the publisher, the editors and the reviewers. Any product that may be evaluated in this article, or claim that may be made by its manufacturer, is not guaranteed or endorsed by the publisher.
