# Supplementary material for: Diagnosis and Stratification of Pseudomonas aeruginosa Infected Patients by Immunochemical Quantitative Determination of Pyocyanin From Clinical Bacterial Isolates
Source: Front Cell Infect Microbiol. 2021 Dec 14;11:786929. doi: 10.3389/fcimb.2021.786929 (PMC8712664; doi:10.3389/fcimb.2021.786929)
Supplement: Supplementary file 1 [file DataSheet_1.pdf]

Supplementary Material

Diagnostic and Stratification of *Pseudomonas aeruginosa* Infected Patients by Immunochemical Quantitative Determination of Pyocyanin from Clinical Bacterial Isolates

Bárbara Rodríguez-Urretavizcaya[1,2], Nuria Pascual[1,2], Carme Pastells[1,2], M. Teresa Martin-Gomez[3], Lluïsa Vilaplana[1,2,*] and M.-Pilar Marco[1,2,]

[1]Nanobiotechnology for Diagnostics (Nb4D), Institute of Advanced Chemistry of Catalonia, IQAC-CSIC, Barcelona, Spain.
[2]CIBER de Bioingeniería, Biomateriales y Nanomedicina, CIBER-BBN, Madrid, Spain
[3]Microbiology Department, Hospital Universitari Vall d'Hebron, Barcelona, Spain.

[*] **Correspondence**:

Lluisa Vilaplana

Phone: + 34 93 4006100. Fax: + 34 93 2045904.

e-mail: lluisa.vilaplana@cid.csic.es

Keywords: **quorum sensing, pyocyanin, ELISA, *Pseudomonas aeruginosa*, monoclonal antibody, diagnostic**

S.1. PYO mAb/PC1-BSA ELISA characterization

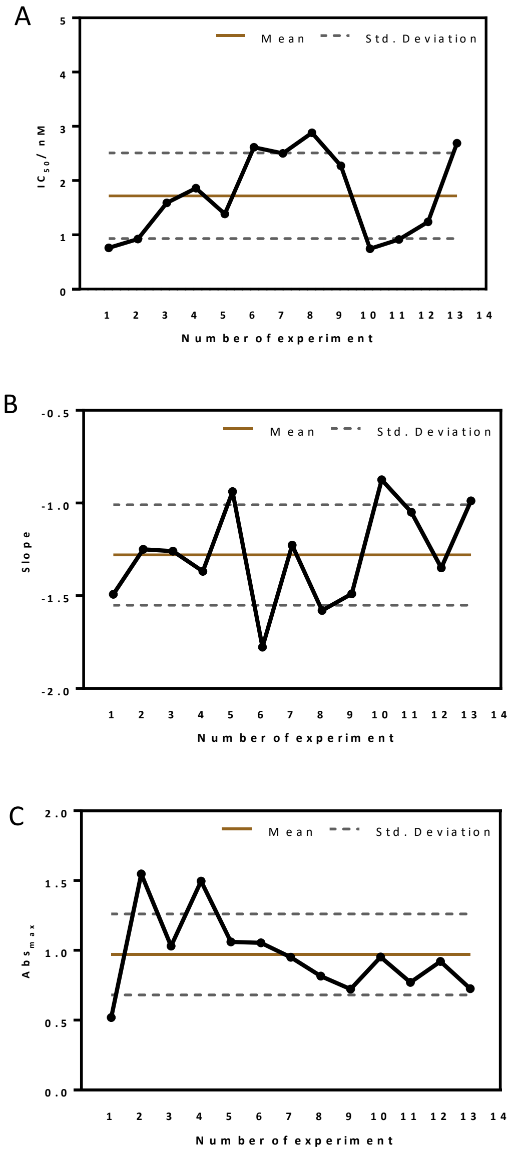

Figure S. 1 (A) IC_{50}, (B) slope and (C) Abs_{max} values of PYO mAb122/PC1-BSA ELISA.13 identical experiments were run in 1/20 diluted MH media using PBST. Results are represented chronologically (in the same year).

S.2. Physicochemical parameters optimization

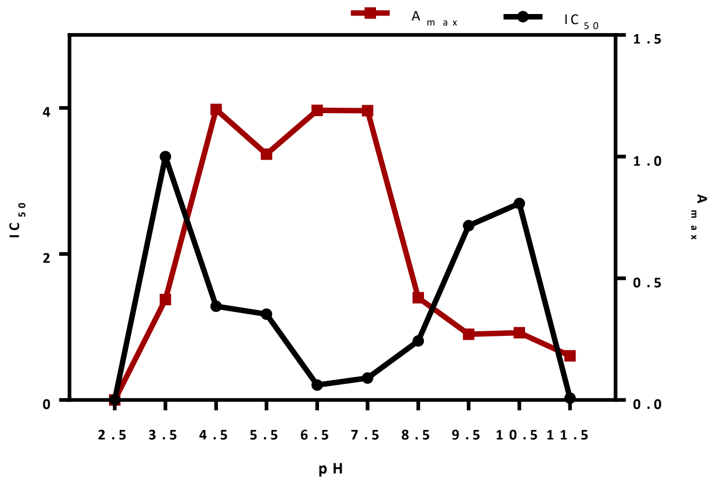

Figure S.2. PYO mAb/PC1-BSA ELISA performance at different pHs ranging from 2.5 to 11.5. All the studies were performed varying the composition of the buffer in the competitive step of the ELISA**.**

S.3. Cross reactivity studies

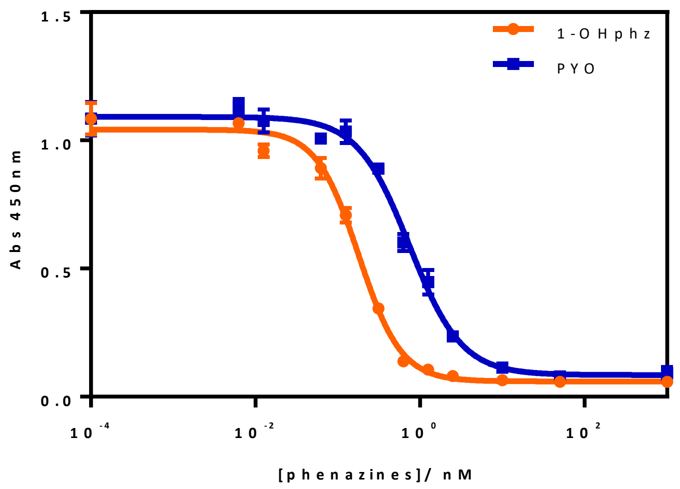

Figure S.3. Cross reactivity study using 1-OHphz and PYO metabolites in buffer under the aforementioned conditions for PYO mAb/ PC1-BSA ELISA. The obtained IC_{50} for 1OHphz and PYO were 0.104 and 0.569 nM, respectively. Each calibration point was measured in triplicates on the same ELISA plate and the results show the average and standard deviation of analysis made on 3 different days.

S.4. Characterization of bacterial isolates

Table S.1. Bacterial growth rates of *P. aeruginosa* bacterial isolates obtained from patients suffering acute or chronic infection, grown in MH media (n=3).

**Acute isolate PAAI20**			**Chronic isolate PACI18**		
T/ h	***OD 600 nm***	***Bact. conc, CFU mL^{-1}***	***T/ h***	***OD 600 nm***	***Bact. conc, CFU mL^{-1}***
0	0.003	3.0 x 10^{5}	0	0.003	3.1 x 10^{5}
1	0.004	4.0 x 10^{5}	1	0.003	6.1 x 10^{5}
2	0.005	1.6 x 10^{6}	2	0.006	1.9 x 10^{6}
3	0.009	3.9 x 10^{6}	12	0.376	3.3 x 10^{9}
4	0.023	3.0 x 10^{7}	13	0.581	1.9 x 10^{9}
5	0.063	6.8 x 10^{7}	14	1.091	1.5 x 10^{9}
6	0.183	3.7 x 10^{8}	15	1.270	2.4 x 10^{12}
7	0.427	5.2 x 10^{11}	16	1.440	2.2 x 10^{12}
8	0.754	1.4 x 10^{12}	17	1.475	1.0 x 10^{13}
9	1.012	5.2 x 10^{12}	24	1.996	5.2 x 10^{12}
24	2.046	1.3 x 10^{13}	48	2.045	3.9 x 10^{11}
48	0.747	2.3 x 10^{12}			

T: time. Bacterial culture turbidity is determined measuring the optical density (OD) at 600 nm.

Table S.2 PYO and 1-OHphz concentrations measured in bacterial isolates from patients infected with *P. aeruginosa*. Bacterial isolates were grown for 16 hours in MH media (n=3).

Number of patient	Isolate name	IRequiv. of PYO /nM	1-OHphz /nM
1	PAAI1	197.24 ± 4.57	22.75 ± 10.27
2	PAAI2	10.24 ± 4.16	2.25 ± 1.63
3	PAAI3	1959.42 ± 543.00	261.49 ± 47.72
4	PAAI4	1672.79 ± 4.47	290.60 ± 18.53
5	PAAI5	4022.66 ± 8.53	330.02 ± 24.17
6	PAAI6	1696.88 ± 42.66	261.92 ± 62.81
7	PAAI7	8.41 ± 2.94	1.65 ± 0.61
8	PAAI8	9.66 ± 0.70	1.69 ± 1.16
9	PAAI9	4970.63 ± 14.75	262.90 ± 55.41
20	PACI1	6.14 ± 1.99	3.00 ± 0.23
21	PACI2	5.82 ± 0.47	3.45 ± 1.16
22	PACI3	9.28 ± 2.35	1.46 ± 0.97
23	PACI4	8.37 ± 0.20	1.45 ± 0.96
24	PACI5	8.71 ± 3.54	1.67 ± 0.46
25	PACI6	9.60 ± 2.33	1.19 ± 0.44
26	PACI7	9.93 ± 1.40	3.69 ± 1.57
27	PACI8	15.60 ± 1.62	5.67 ± 1.32
-	PAO1	3535.12 ± 618.45	436.45 ± 41.38
10	PAAI10	27.18 ± 8.10	10.24 ± 1.89
11	PAAI11	29.02 ± 2.90	1.75 ± 0.93
12	PAAI12	179.26 ± 3.67	14.82 ± 2.50
13	PAAI13	510.57 ± 13.43	28.06 ± 0.51
14	PAAI14	106.54 ± 14.94	80.25 ± 5.62
15	PAAI15	8235.99 ± 993.75	217.65 ± 2.16
16	PAAI16	6195.56 ± 602.20	156.00 ± 13.06
17	PAAI17	22.34 ± 4.23	2.29 ± 1.14
18	PAAI18	22.14 ± 6.79	5.45 ± 0.96
19	PAAI19	4822.15 ± 241.55	137.61 ± 3.47
28	PACI9	6.55 ± 2.75	2.43 ± 0.00
29	PACI10	205.77 ± 5.13	23.09 ± 2.59
30	PACI11	*n.d.*	0.62 ± 0.30
31	PACI12	4.91 ±0.64	0.91 ± 0.35
32	PACI13	5.74 ± 0.70	0.71 ± 0.54
33	PACI14	3.73 ± 0.76	1.18 ± 0.34
34	PACI15	2.04 ± 0.11	2.67 ± 0.08
35	PACI16	0.96 ± 0.33	4.02 ± 0.42
36	PACI17	2.91 ± 0.60	2.45 ± 0.95
37	PACI18	0.93 ± 0.34	1.88 ± 0.07

PA = *P. aeruginosa*; AI = acute isolate; CI= chronic isolate; *n.d.*= not detected
